# Supplementary material for: Climate change modulates the stratospheric volcanic sulfate aerosol lifecycle and radiative forcing from tropical eruptions
Source: Nat Commun. 2021 Aug 12;12:4708. doi: 10.1038/s41467-021-24943-7 (PMC8360950; doi:10.1038/s41467-021-24943-7)
Supplement: Supplementary file 1 — Supplementary Information [file 41467_2021_24943_MOESM1_ESM.pdf]

Supplementary Information for "Climate change modulates the stratospheric volcanic sulfate aerosol lifecycle and radiative forcing from tropical eruptions"

Thomas J. Aubry[*1,2], Johnny Staunton-Sykes[3], Lauren R. Marshall[3], Jim Haywood[4,5], Nathan Luke Abraham[3,6], Anja Schmidt[1,3]

[1] Department of Geography, University of Cambridge, Cambridge, UK

[2] Sidney Sussex College, Cambridge, UK

[3] Department of Chemistry, University of Cambridge, Cambridge, UK

[4] College of Engineering Mathematics and Physical Sciences, University of Exeter, UK

[5] Met Office Hadley Centre, Exeter, UK

[6] National Centre for Atmospheric Science, UK

*Correspondence and requests for materials should be addressed to T.J.A. (email:ta460@cam.ac.uk)

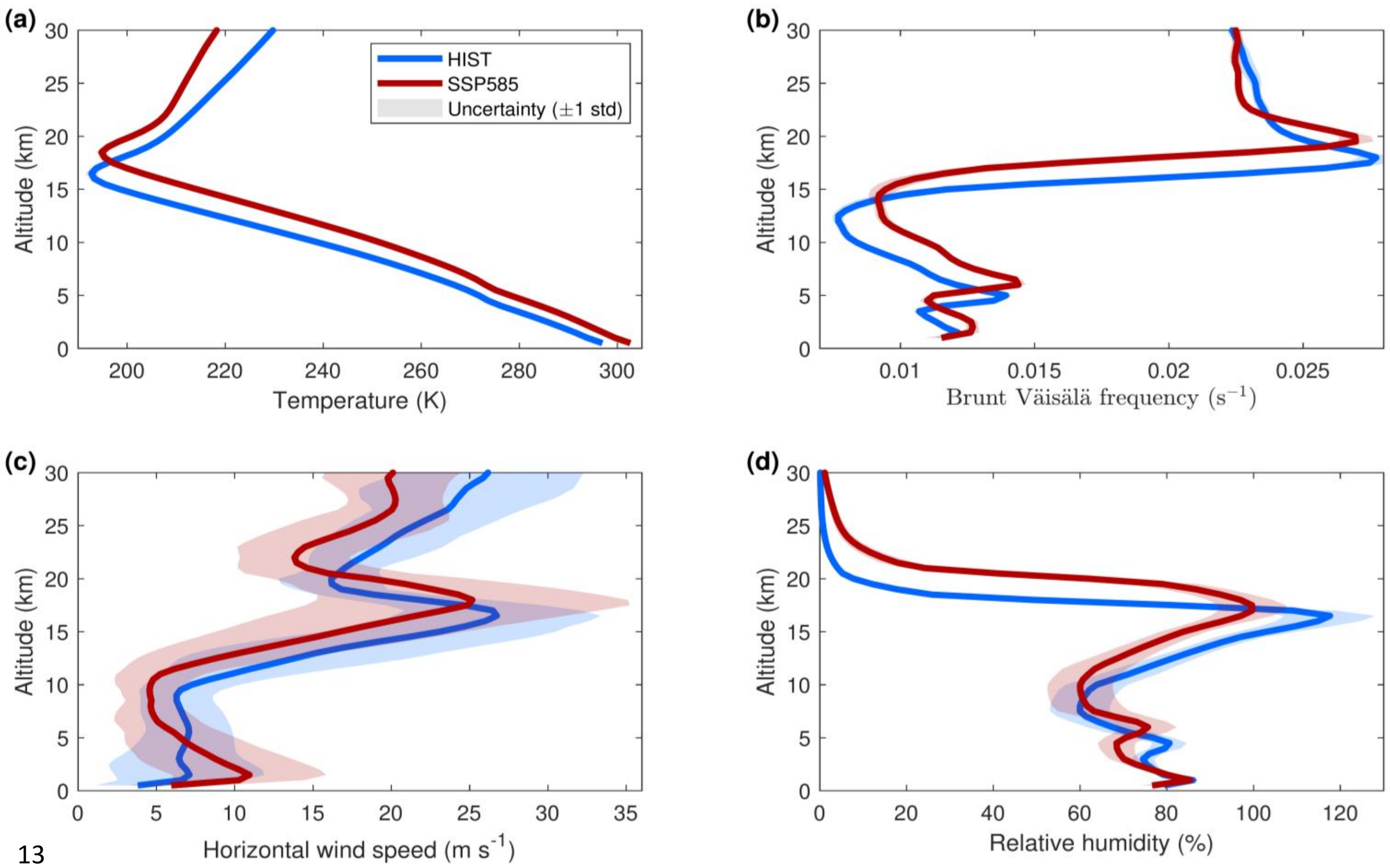

Supplementary Figure 1: Atmospheric conditions used as input to the eruptive column model. Profiles of temperature (a), Brunt-Väisälä frequency (b), horizontal wind speed (c) and relative humidity (d) simulated by UKESM1 at the location of Mt Pinatubo (15.1°N,120.4°E) on July 1st in our control runs. Profiles are shown for the HIST (blue) and SSP585 (red) scenarios, and shadings show one standard deviation across the 20 years of each control run.

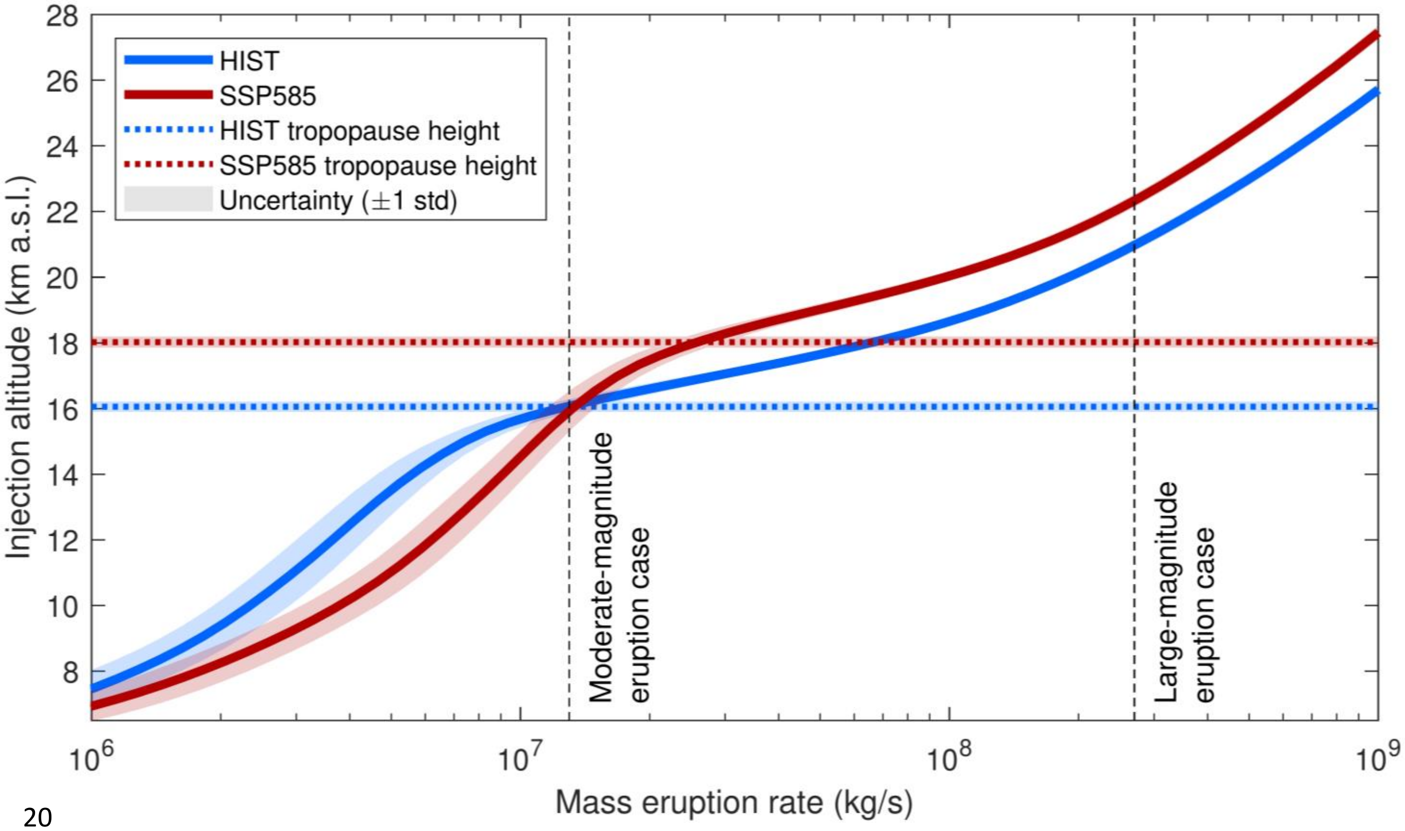

Supplementary Figure 2: Injection altitude predicted by the 1D eruptive column model used in this study. Results are shown for mass eruption rates (also referred to as eruption intensity) between 10^6 and 10^9 kg s^{-1}, and using atmospheric profiles shown in Supplementary Figure 1. Blue refer to the HIST scenario and red refer to the SSP585 scenario, with shadings showing one standard deviation across the 20 set of atmospheric profiles. Horizontal dashed lines show the tropopause height, and black vertical dashed line show mass eruption rates corresponding to our moderate-magnitude (1.3 10^7 kg s^{-1}) and large-magnitude (2.7 10^8 kg s^{-1}) eruption cases.

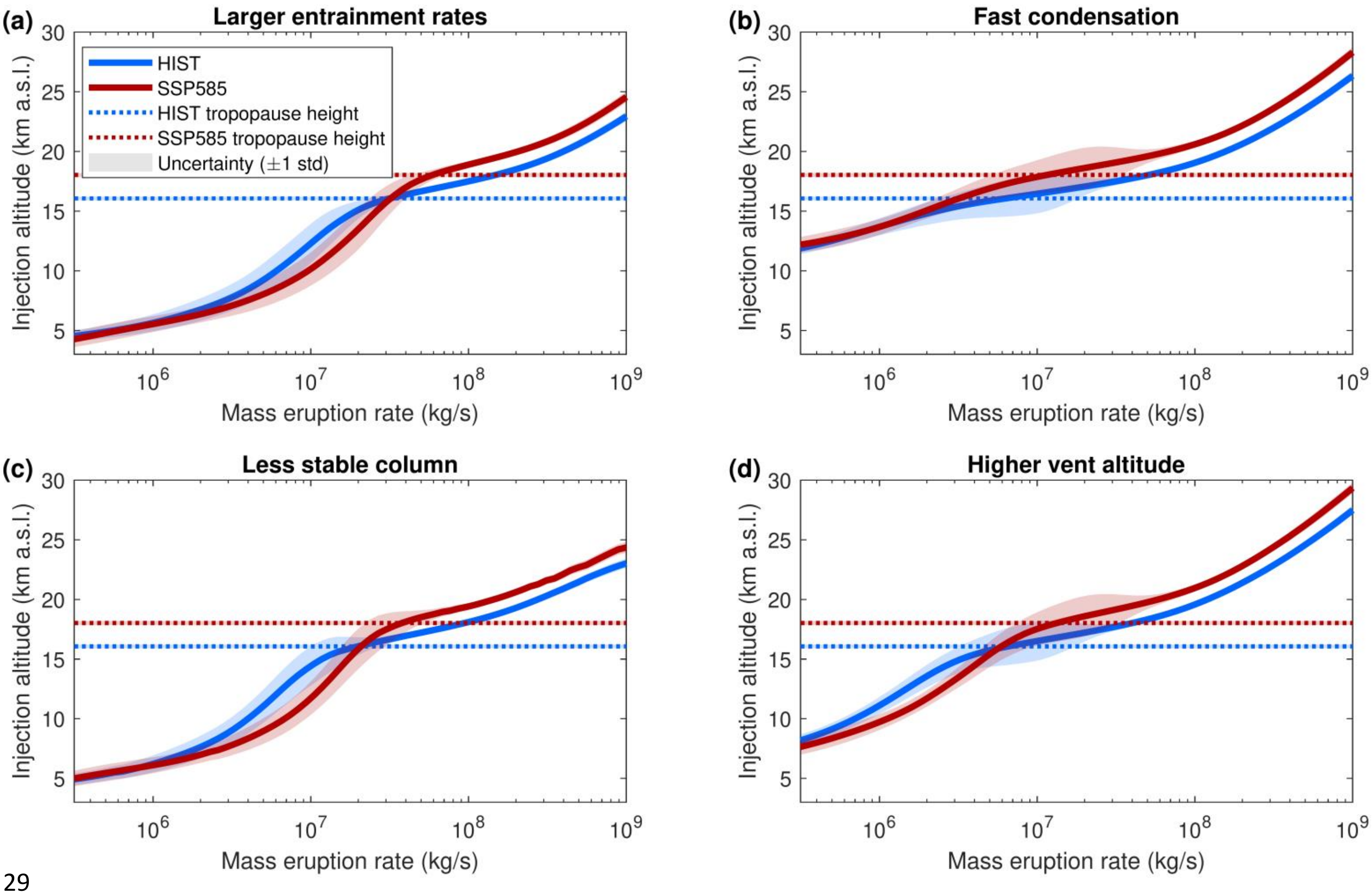

Supplementary Figure 3: Sensitivity of plume modelling results to source and model parameters. Same as Supplementary Figure 2, but using different inputs or eruptive column model parameters than those used in Supplementary Figure 2 that were applied to infer injection heights in UM-UKCA simulations. In panel (a), we used radial and wind entrainment rates of 0.12 and 0.75, instead of 0.1 and 0.25 in Supplementary Figure 2. In panel (b), we used a condensation rate of 10^{-2} s^{-1} instead of 10^{-6} s^{-1} in Supplementary Figure 2. In panel (c), we used an injection gas content, temperature and vent radius injection velocity ratio of 0.02 wt.%, 800 C and 0.004 instead of 0.05 wt.%, 1100 C and 0.002 in Supplementary Figure 2. In panel (d), we used a vent altitude of 3500 m instead of 1500 m in Supplementary Figure 2.

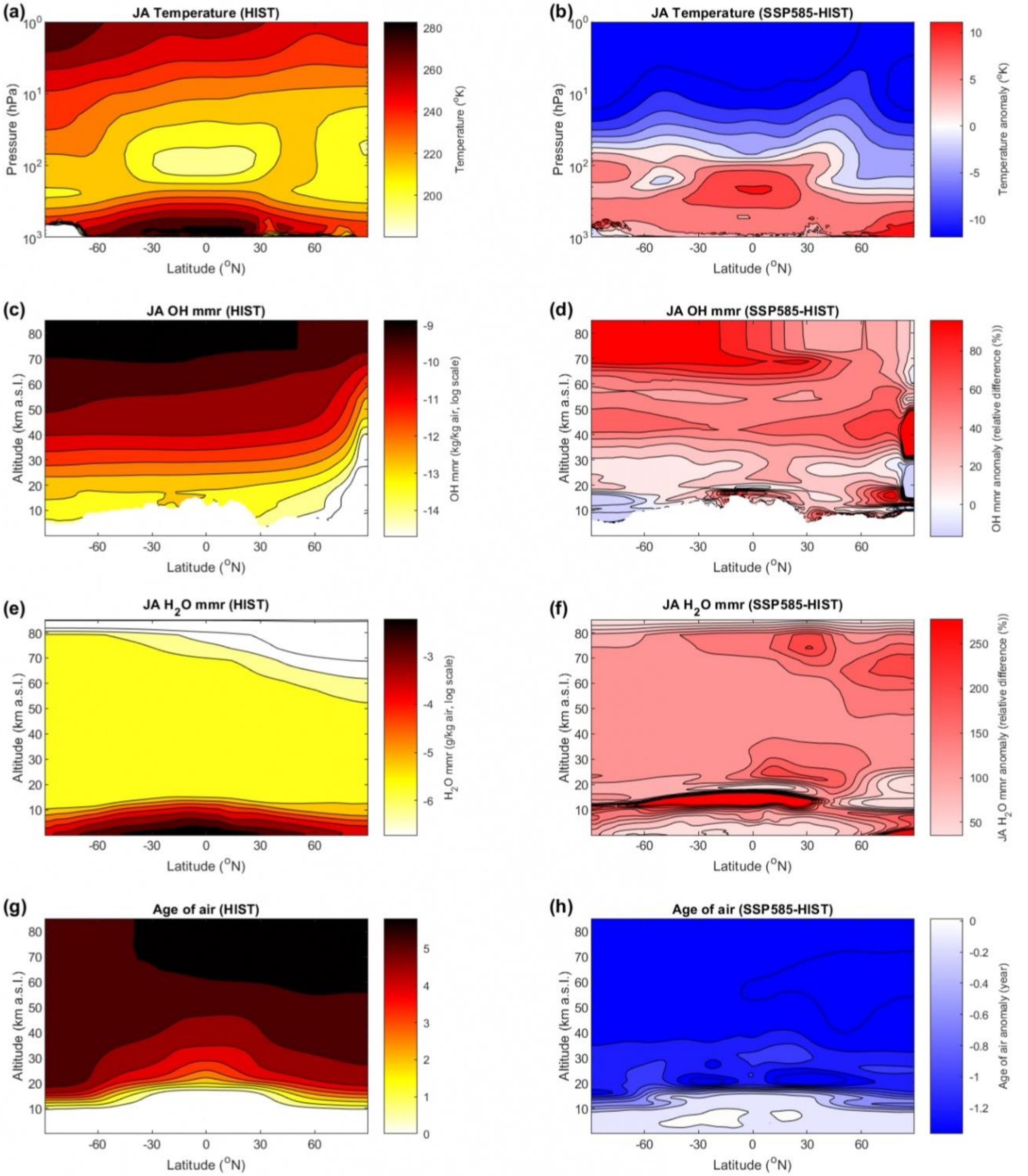

Supplementary Figure 4: Background state of key climate variables in our control simulations. Zonal mean values of July-August (JA) temperature (a,b), JA OH mass mixing ratio (mmr, c,d), JA H_2O mmr (e,f)

and yearly mean age of air (g,h) in our control runs (see Methods). Left panels (a,c,e,g) show values for HIST, right panels (b,d,f,h) shows the difference between SSP585 and HIST.

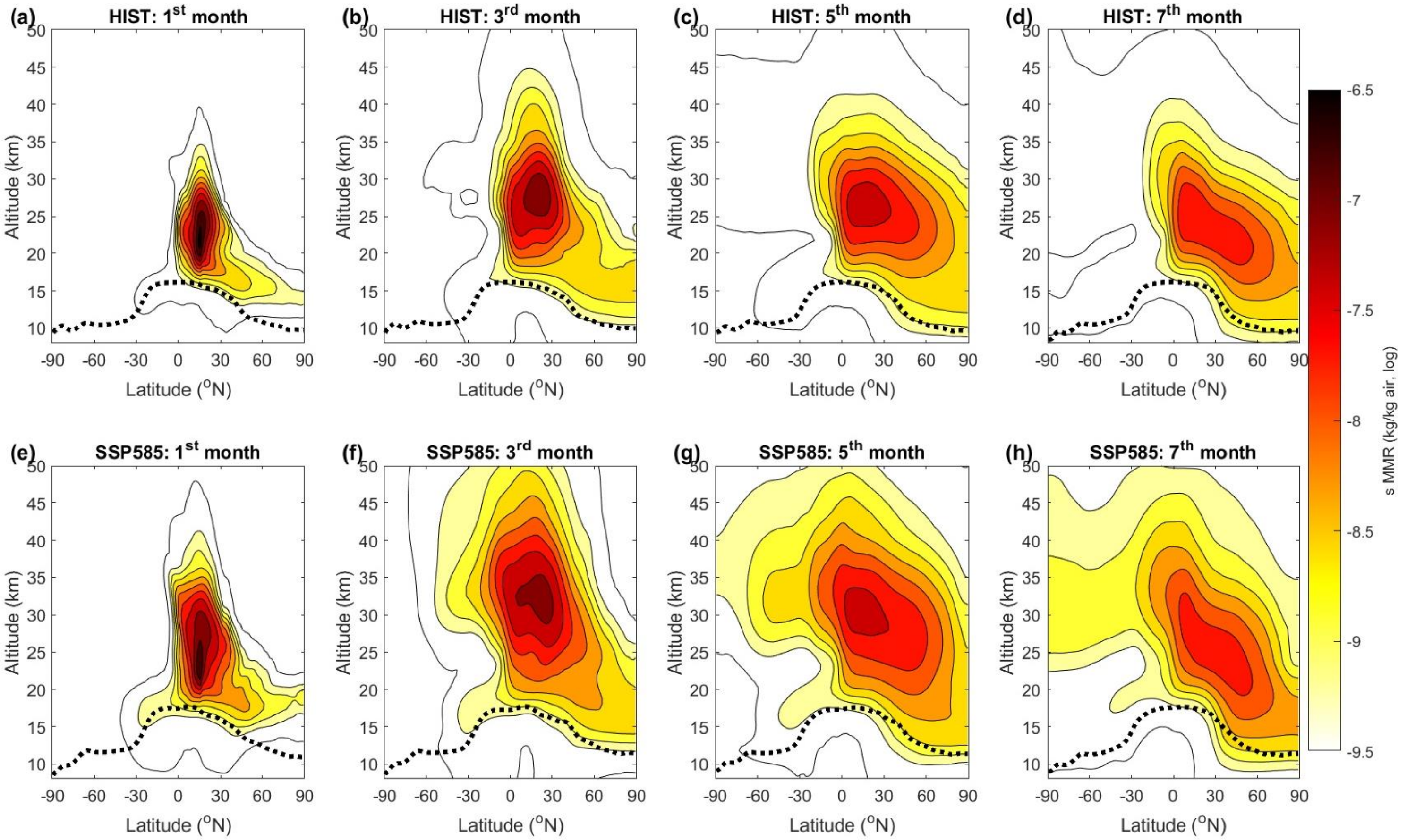

Supplementary Figure 5: Initial sulfur distribution for the large-magnitude eruption. Same as Figure 3, but showing S mass mixing ratios on a log scale for the large-magnitude eruption and every two months for the first seven post-eruption months.

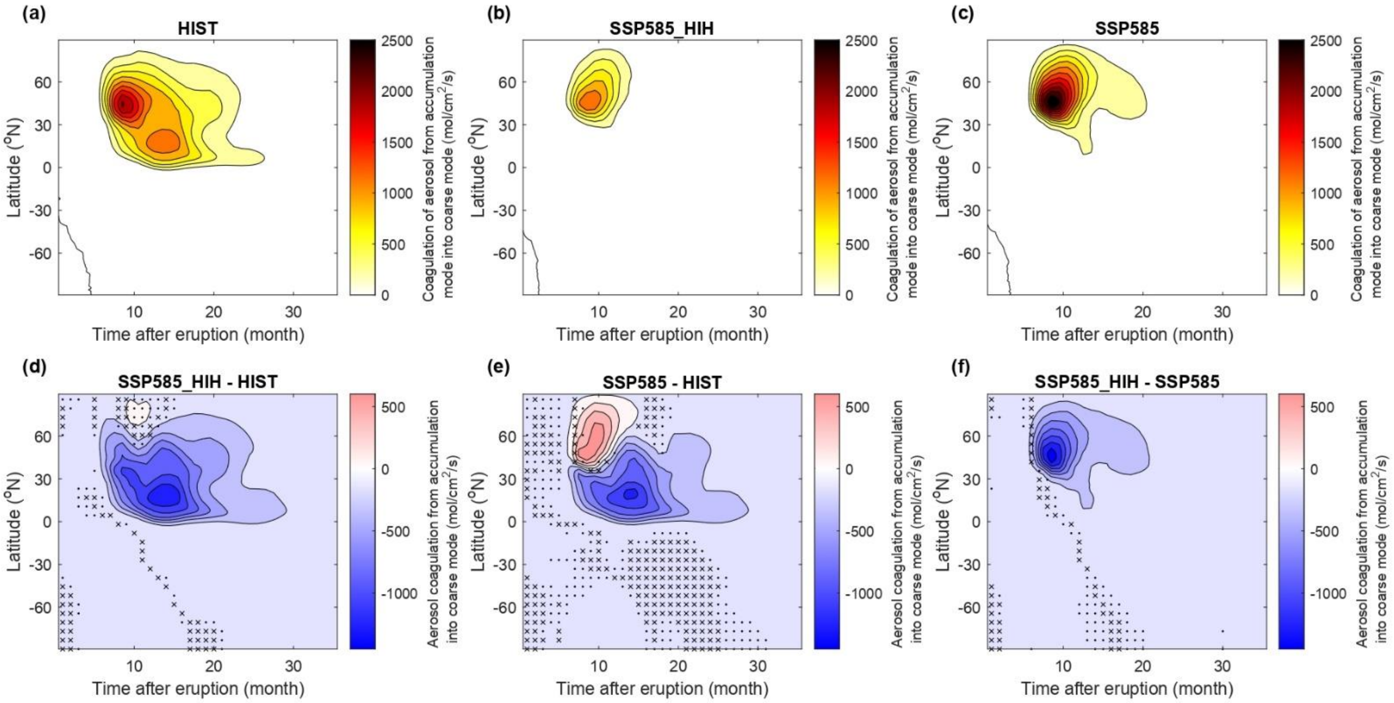

Supplementary Figure 6: Time-latitude evolution of the aerosol coagulation rate (mol cm^{-3} s^{-1}) for the large-magnitude eruption. Same as Figure 6, but showing the rate of coagulation of aerosol particles from the accumulation mode to form aerosol particles in the coarse mode.

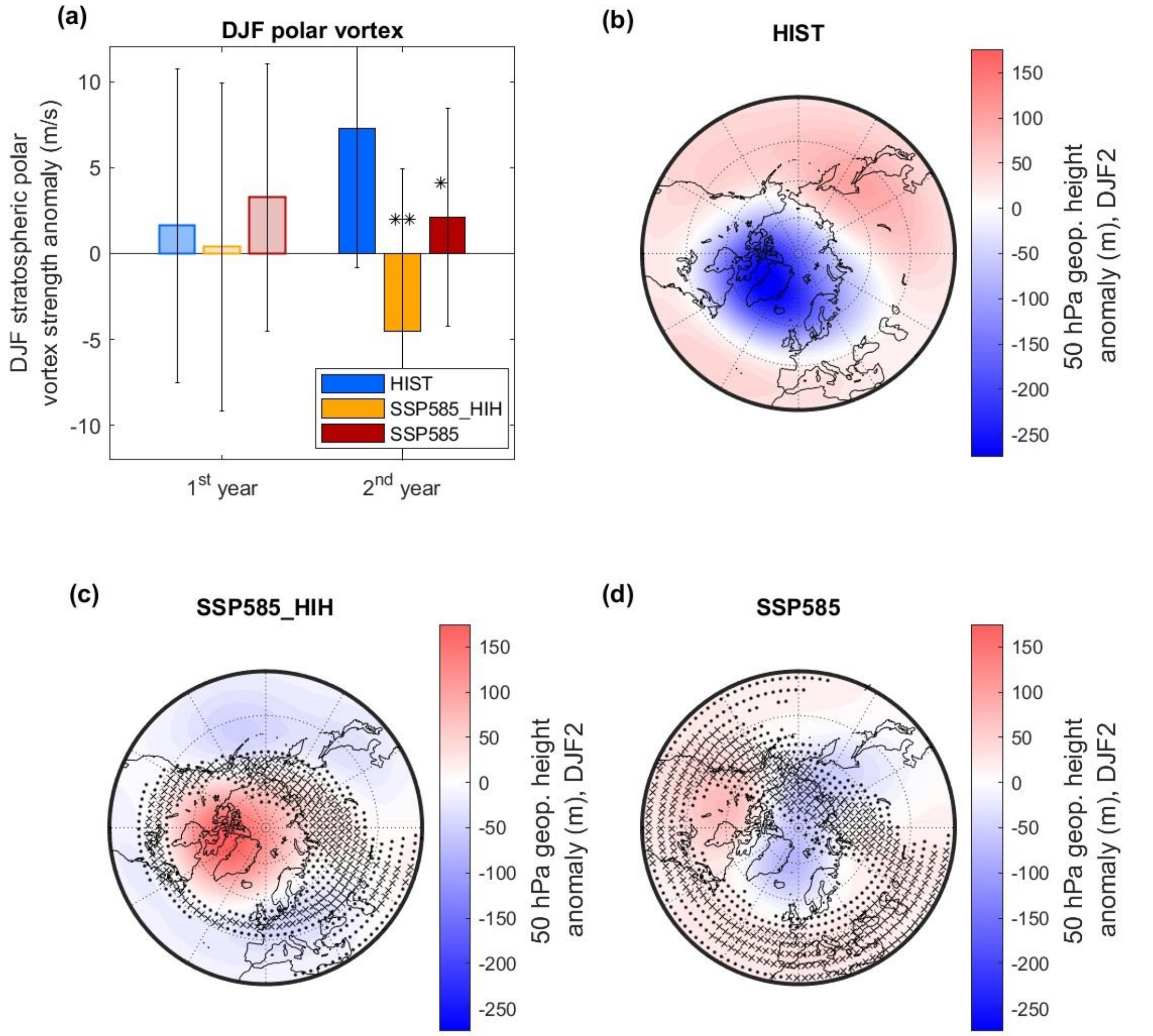

Supplementary Figure 7: Polar vortex response in our simulations for the large-magnitude eruption. Panel (a): Winter (DJF) stratospheric polar vortex strength anomaly for the 1st and 2nd post-eruption years. The polar vortex strength was defined as the zonal mean wind speed averaged over 55-65°N and 1-30hPa. Error bars show one standard deviation across the ensemble; a single (respectively double) star indicates that the change relative to the HIST experiment is significant at the 80% (respectively 95%) level.

Panels b-d: Geopotential height anomaly at 50hPa over the Northern hemisphere averaged over the first post-eruption winter (DJF) for the HIST (b), SSP585_HIH (c) and SSP585 (d) scenarios. Dots highlight areas where changes are not significant at the 95% and crosses highlight areas where the difference is not significant at the 80% level.

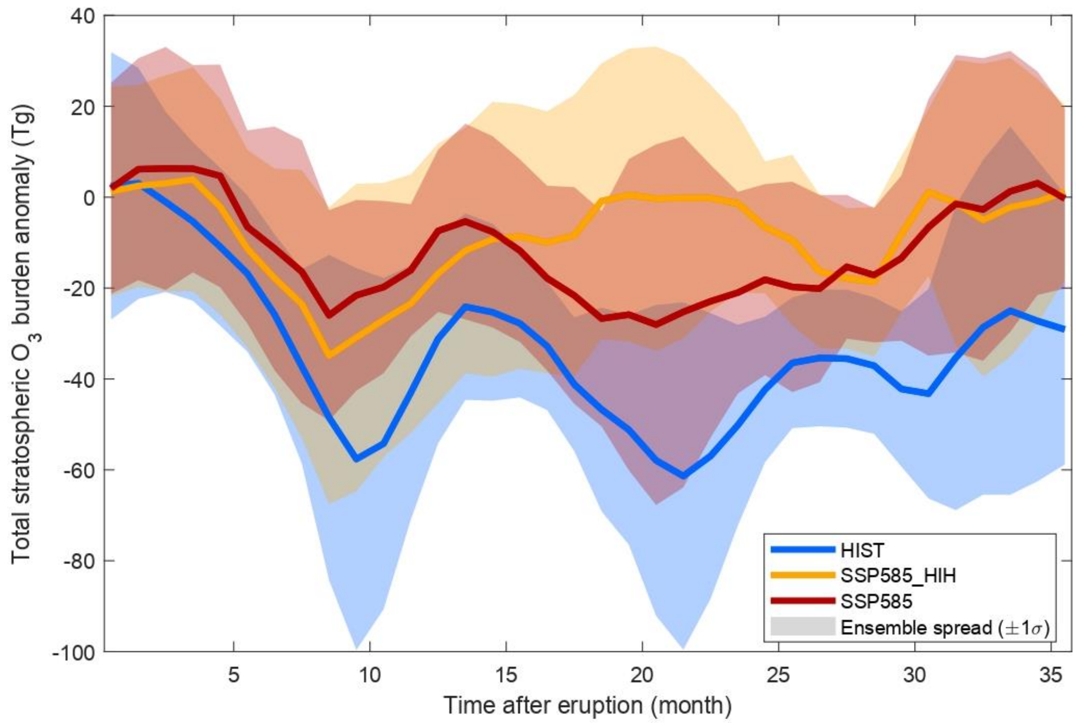

Supplementary Figure 8: Total stratospheric ozone (O_3) burden anomaly time series in our simulations for the large-magnitude eruption.

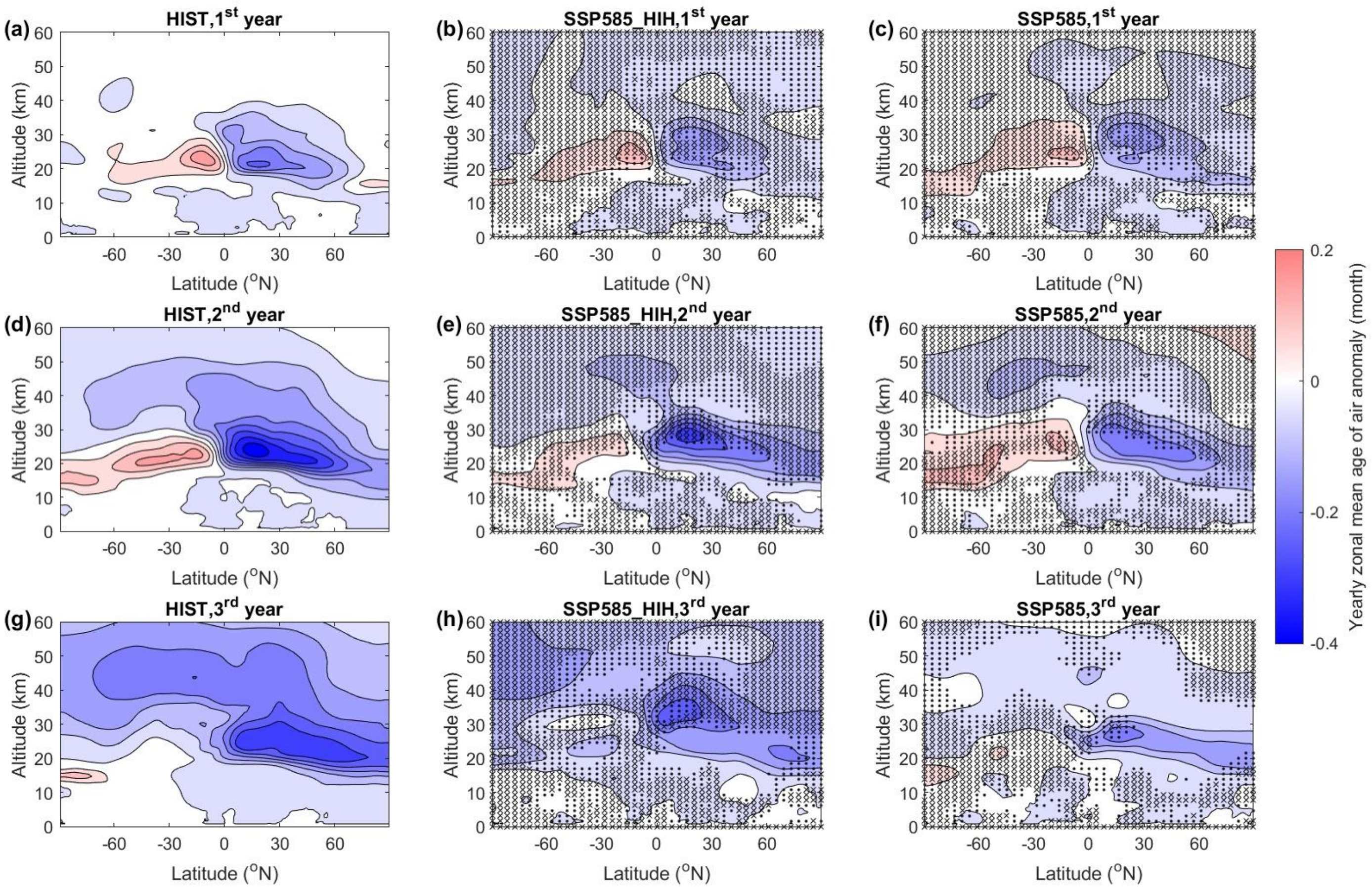

Supplementary Figure 9: Zonal annual mean age of air anomaly for the 1st (a-c), 2nd (d-f) and 3rd (g-i) post-eruption year for the three scenarios considered in this study for the large-magnitude eruption. In panels b, c, e, f, h and I, dots highlight areas where changes relative to the HIST scenario are not significant at the 95% and crosses highlight areas where the difference is not significant at the 80% level.
